# Latent profile analysis of fatalism and its influencing factors among community-dwelling disabled elderly individuals

**DOI:** 10.3389/fpsyg.2025.1507591

**Published:** 2025-01-28

**Authors:** Jinlei Du, Xiaoling Wu, Qiyu Zhang, Yuanxia Wang, Yao Chen, Chencong Nie

**Affiliations:** Zigong Fourth People's Hospital, Zigong, China

**Keywords:** community, disabled elderly, fatalism, profile analysis, influence factor

## Abstract

**Objective:**

This study aims to explore the latent profiles of fatalism among community-dwelling disabled elderly individuals and identify the key factors influencing these profiles. The findings will provide valuable insights for formulating tailored care management strategies for this population.

**Design:**

A cross-sectional survey study.

**Methods:**

A random sampling approach was used to survey disabled elderly individuals residing in 109 communities across eight urban districts in Sichuan Province. Data were collected through a general information questionnaire and a Fatalism Scale. Latent profile analysis was performed to identify distinct fatalism profiles, and multivariate unordered regression analysis was conducted to assess their influencing factors.

**Results:**

Three distinct latent profiles of fatalism were identified: high fatalism and pessimism tendency (35.6%), moderate fatalism and low optimism tendency (9.6%), and low fatalism with relative optimism tendency (54.8%). Multivariate analysis revealed that living arrangements, number of children, educational level, duration of disability, and self-reported economic stress were significant factors influencing these fatalism profiles.

**Conclusion:**

There is significant heterogeneity in fatalism among community-dwelling disabled elderly individuals. Caregivers and healthcare managers can develop more precise and personalized management strategies by considering the different latent profiles and their associated influencing factors.

## 1 Introduction

With the global aging population accelerating, the number of disabled elderly individuals is steadily increasing (Keshavarz et al., 2023). Current statistics indicate that there are over 100 million disabled elderly people worldwide, with more than 40 million residing in China, representing 18.3% of the total elderly population (Bao et al., 2022). As their ability to perform daily activities declines, disabled elderly individuals often face considerable physical and psychological challenges (Ma et al., 2022), resulting in negative emotions such as anxiety, depression, apathy, and pessimism, which severely diminish their quality of life. Fatalism, a common worldview characterized by the belief in the uncontrollability of fate, plays a significant role when individuals encounter major life changes and challenges (Keller et al., 2024). However, an excessive sense of fatalism among disabled elderly individuals may lead to passive acceptance of adversity, exacerbating negative coping mechanisms (Sharrief et al., 2017).

The community serves not only as the primary living environment for elderly individuals but also as a vital source of social interaction, recreation, access to resources, and social support (Deardorff et al., 2022). Despite this, current psychological research on disabled elderly individuals in community settings predominantly focuses on assessing mental health through overall scale scores, often neglecting the nuanced differences within patient subgroups (Zivoder et al., 2019). Latent profile analysis (LPA), a person-centered approach, allows for the identification of underlying characteristics by analyzing individual responses across various dimensions of a scale (Băjenaru et al., 2022). This method helps elucidate the distribution of distinct subgroups within a population.

Therefore, this study employs LPA to classify the fatalism characteristics among community-dwelling disabled elderly individuals and explore the differences across these latent types. The findings aim to assist clinical healthcare professionals in developing targeted intervention strategies tailored to the needs of different subgroups.

## 2 Subjects and methods

### 2.1 Study population

From July 10 to July 20, 2024, this study targeted disabled elderly residents from 109 communities across eight cities in Sichuan Province, namely Chengdu, Deyang, Zigong, Neijiang, Yibin, Luzhou, Leshan, and Nanchong. The required sample size was estimated using the formula $N=\frac{{\left(Z_{\alpha /2}\cdot \sigma \right)}^{2}}{E^{2}}$ , where $Z_{\alpha /2}$ represents the z-score for a 95% confidence interval (approximately 1.96), *σ* is the population standard deviation, and E is the desired width of the confidence interval. Based on a preliminary survey of 30 disabled elderly individuals in local communities, an estimated standard deviation σ ≈ 10 was calculated. To achieve a high level of precision with a confidence interval width of ±1 unit, indicating that actual scale scores would vary within ±1 unit at a 95% confidence level, the required sample size was determined to be 385. Adjusting for a potential 10% rate of invalid questionnaires, the final sample size was set at 424 disabled elderly individuals.

To ensure sample representativeness and the scientific validity of the results, a random sampling approach was used. The number of participants selected from each community was dynamically adjusted based on community size and the number of disabled elderly residents. Each community selected between 3 and 10 individuals randomly, based on these factors.

Inclusion Criteria: Aged 60 years or older, Diagnosed with disability according to the Barthel Index (score less than 100), Normal communication ability with no impairment of consciousness, Voluntarily participated in the study with informed consent. Exclusion Criteria: Currently participating in other psychological-related surveys or studies.

### 2.2 Ethical approval

This study adheres to the National Code of Ethical Conduct for Human Research (2016) and has received approval from the Ethics Committee of Zigong Fourth People’s Hospital (Approval No: EC-2023-073). All procedures were conducted in accordance with the Declaration of Helsinki. Informed consent was obtained from all participants prior to their involvement in the study.

### 2.3 Survey instruments

#### 2.3.1 General information questionnaire

The general information questionnaire was developed by the research team based on a comprehensive literature review and expert consultations. It includes demographic and disease-related data. Demographic information encompasses gender, age, marital status, and number of children. Age categories are defined as follows: 60–69 years as young-old, 70–79 years as middle-old, and 80 years and above as old-old, as per the China Population Science Dictionary. Marital status is categorized into married and non-married, with the non-married category including divorced, widowed, or never married individuals. The primary caregiver category includes options such as “others,” which typically refers to caregivers like nannies or fellow patients.

Disease-related data include the primary disabling condition, the number of comorbidities, and the degree of disability. Comorbidities refer to additional systemic diseases beyond the primary disabling condition. Disability degree is assessed using the Barthel Index rating scale.

#### 2.3.2 Barthel index rating scale

The Barthel Index, developed by Mahoney in 1965, assesses 10 areas of daily living. A lower score indicates greater disability, with a total score of 100. Scores ≤40 indicate severe disability, 41–60 indicate moderate disability, 61–99 suggest mild disability, and 100 denotes full independence in daily living (Izumi et al., 2024).

#### 2.3.3 Fatalism scale

The Fatalism Scale (Shen et al., 2009), developed by Shen and colleagues in 2009, includes three dimensions: Predetermination (items 1–10), Luck (items 11–14), and Pessimism (items 15–20). It employs a Likert 5-point scale ranging from 1 (“strongly disagree”) to 5 (“strongly agree”), with higher scores indicating stronger fatalistic beliefs. The scale demonstrates good reliability and validity, with a Cronbach’s alpha coefficient of 0.88 and a content validity index of 0.89.

### 2.4 Data collection methods

Prior to the study, team members briefed community healthcare center staff on the study’s background and methods. The principal investigator assigned unique identification numbers to each survey questionnaire based on study regions. Questionnaires were distributed via mail or delivery to community healthcare centers, where participants completed them independently and anonymously. Each participant could fill out the survey only once. Completed surveys were collected only if all sections were fully answered. After data collection, regional supervisors gathered the completed surveys and sent them to the central research team.

### 2.5 Quality control

To ensure data accuracy, a research team was formed before the study’s initiation. Each healthcare institution appointed a survey supervisor who received standardized training and was responsible for quality control and data collection within their region. Two researchers reviewed all returned questionnaires, cross-checking them against the expected number for each region. Questionnaires with apparent biases or multiple corrections were excluded. The analysis and interpretation of data were reviewed by experts in statistics to ensure the results accurately reflected clinical significance.

### 2.6 Statistical methods

Exploratory latent profile analysis (LPA) was performed using Mplus 8.3 software to classify community-dwelling disabled elderly individuals based on their fatalism scores. Differences in social factors and demographic characteristics among fatalism categories were compared using chi-square tests, one-way ANOVA, or rank-sum tests.

Variables showing statistically significant differences in univariate analyses were included as independent variables in a multinomial logistic regression analysis, with fatalism categories as the dependent variable. This analysis aimed to explore factors influencing the categorization of fatalism among the elderly.

Model fit indices included the Akaike Information Criterion (AIC), Bayesian Information Criterion (BIC), Adjusted Bayesian Information Criterion (aBIC), and Entropy, along with the Lo–Mendell–Rubin (LMR) test and the bootstrapped likelihood ratio test (BLRT). Lower AIC, BIC, and aBIC values indicate better model fit, and an Entropy value close to 1.0 suggests better predictive accuracy. An Entropy value around 0.8 indicates classification accuracy exceeding 90% (Ford et al., 2022). Significant *p*-values for LMR and BLRT indicate that the model with k classes is significantly better than the model with k − 1 classes. Statistical significance was set at *p* < 0.05.

## 3 Results

### 3.1 General data of community-dwelling disabled elderly individuals

A total of 527 questionnaires were collected for this study. Of these, 480 were randomly selected, and 469 were valid, yielding an effective response rate of 97.70%. Among the 469 community-dwelling disabled elderly individuals, there were 226 males and 243 females. The distribution of disability severity was as follows: 185 individuals with mild disability, 186 with moderate disability, and 98 with severe disability. The mean score on the Fatalism Scale was 48.48 ± 10.23. The dimension scores were: Predetermination 18.65 ± 4.77, Luck 12.62 ± 3.46, and Pessimism 17.20 ± 4.78.

### 3.2 Latent profile analysis results of fatalism among community-dwelling disabled elderly individuals

**Figure 1:**
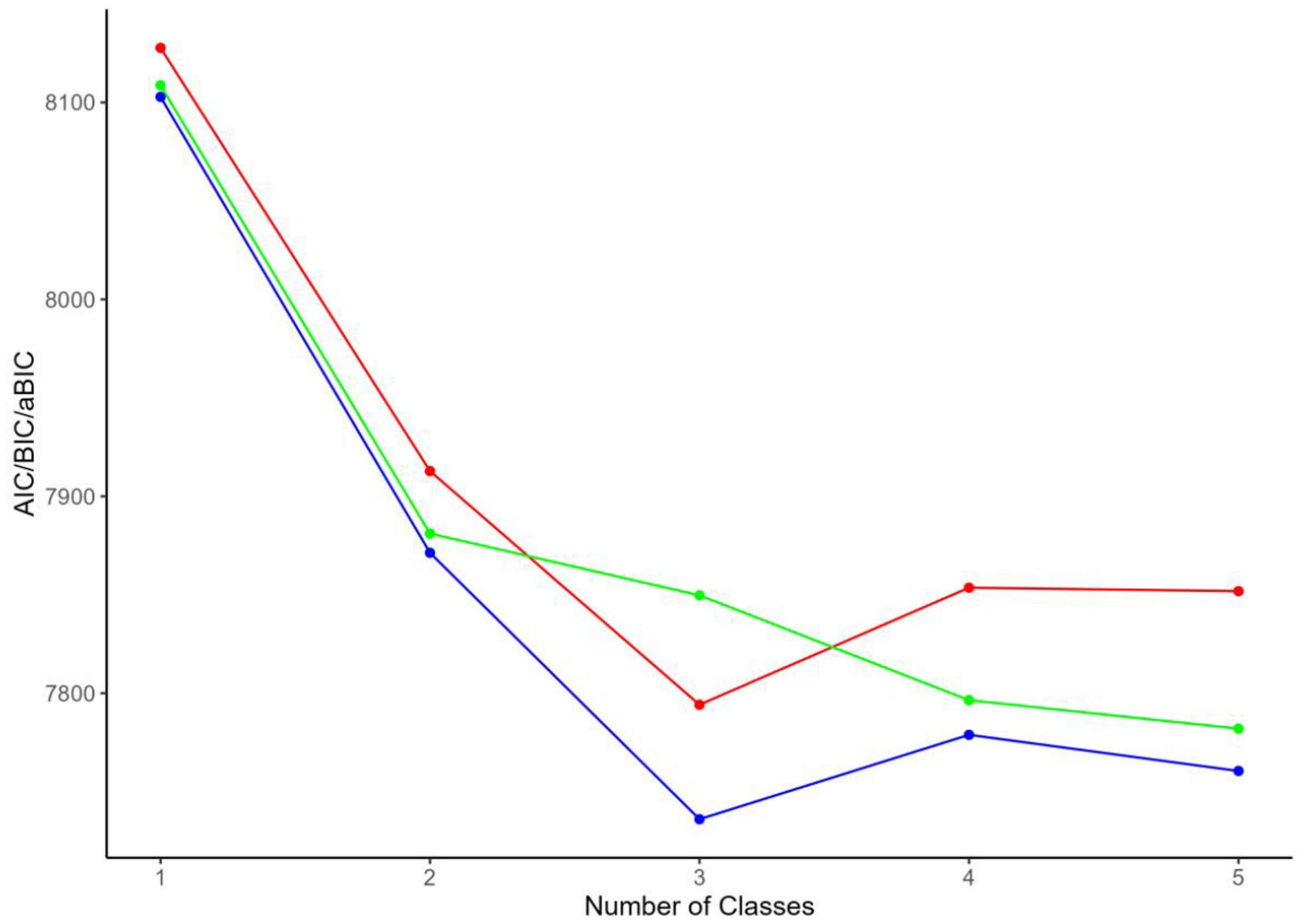
Elbow plot for model selection in latent profile analysis of fatalism in community-dwelling disabled elderly.

Latent profile analysis (LPA) was conducted based on the Fatalism Scale scores. Five potential profile models were assessed using AIC, BIC, aBIC, Entropy, LMR, and BLRT to evaluate the heterogeneity of fatalism among community-dwelling disabled elderly individuals. The results of the LPA are summarized in Table 1. As the number of profiles increased, AIC, BIC, and aBIC showed a consistent decreasing trend. Among the tested models, the three-profile solution was identified as the optimal fit. Compared to Models 2, 4, and 5, the three-profile model not only achieved the highest Entropy value but also demonstrated statistical significance in both LMR and BLRT (*p* < 0.05). Additionally, although the four-class and five-class models exhibited lower AIC and BIC values, they resulted in smaller class sizes, which could undermine the interpretability and practical relevance of the latent profiles. Detailed information is provided in Table 1 and Figure 1.

**Table 1:** Fit indices for different latent profile analysis models (*n* = 469).

Model	AIC	BIC	aBIC	Entropy	*P*-value		Class probability
					LMR	BLRT	
1	8102.806	8127.709	8108.666	–	–	–	
2	7871.307	7912.813	7881.075	0.776	<0.001	<0.001	0.356/0.643
3	7736.048	7794.157	7849.724	0.872	<0.001	<0.001	0.356/0.096/0.548
4	7778.884	7853.595	7796.467	0.823	0.001	<0.001	0.185/0.194/0.460/0.159
5	7760.541	7851.854	7782.030	0.792	0.229	<0.001	0.166/0.104/0.198/0.462/0.068

The analysis identified three latent profiles:

*Class 1*: Comprising 167 individuals (35.6%). This group exhibited high scores on the Predetermination and Pessimism dimensions of the Fatalism Scale, reflecting a strong belief in the uncontrollability of fate and pronounced pessimism. Individuals in this class tend to perceive many events as predestined and maintain a generally negative outlook on the future. Their Luck dimension scores were moderately high, indicating some expectation of good fortune, but the overall impact of pessimism was significant. Thus, this group is named as “High Fatalism and Pessimistic Tendency.”
*Class 2*: Consisting of 45 individuals (9.6%). This group demonstrated moderately high scores on the Predetermination dimension, signifying a moderate level of fatalistic belief, though less intense than Class 1. The Luck dimension scores were low, indicating minimal expectation of good fortune and skepticism about luck. Pessimism scores were moderately low, suggesting some negative emotions but a less severe outlook overall. This group is named as “Moderate Fatalism and Low Optimism Tendency.”
*Class 3*: Including 257 individuals (54.8%). This class showed lower scores on the Predetermination dimension, reflecting greater trust in personal control over fate and belief in the influence of choices and efforts on life outcomes. Luck dimension scores were moderate, suggesting an openness to good fortune. Pessimism scores were also moderate but lower compared to the other classes. This group is named as “Low Fatalism and Relatively Optimistic Tendency.”

**Figure 2:**
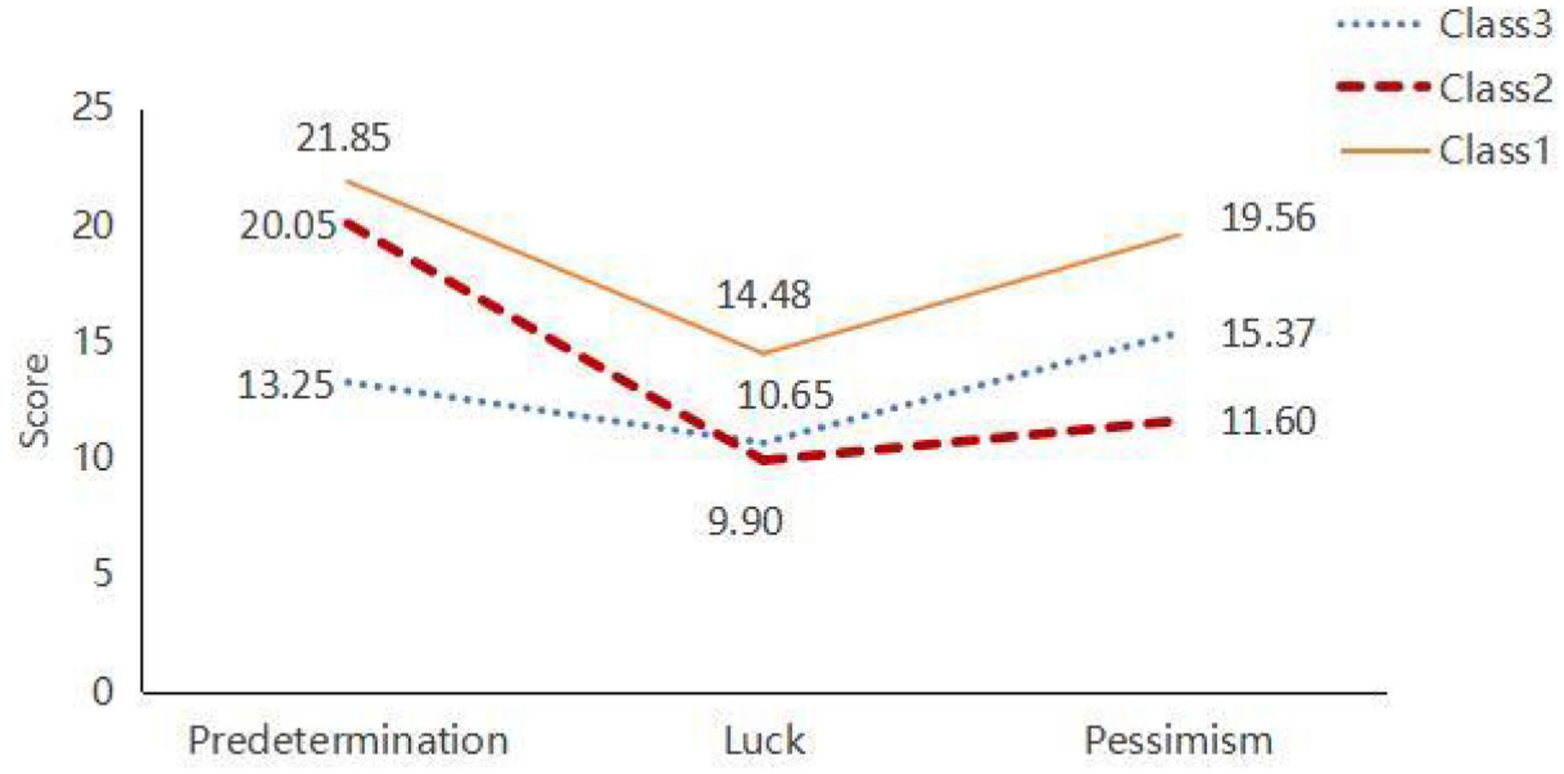
Latent profiles of fatalism in community-dwelling disabled elderly.

The scores for each dimension of the Fatalism Scale for Class 1, Class 2, and Class 3 are illustrated in Figure 2.

### 3.3 Univariate analysis results

Univariate analysis was conducted with the three latent profiles of fatalism among community-dwelling disabled elderly individuals as dependent variables, and demographic and disease-related data as independent variables. The analysis revealed that several factors significantly influenced the differences in fatalism profiles. These factors include: Number of Children, Payment Method for Medical Expenses, Educational Level, Duration of Disability, Living Situation, Primary Caregiver, Self-Rated Family Harmony, Self-Rated Economic Pressure. Each of these factors was found to have a significant impact on the variation in fatalism profiles, indicating that they play a crucial role in shaping the fatalistic views of disabled elderly individuals living in the community. The detailed results are presented in Table 2.

**Table 2:** Univariate analysis of fatalism profiles in community-dwelling disabled elderly individuals.

Options		Class 1 *n* = 167,35.6%	Class 2 *n* = 45,9.6%	Class 3 *n* = 257,54.8%	Statistics	*P*
Gender No (%)	Male	81 (48.51)	17 (37.77)	128 (49.80)	2.229^a^	0.328
	Female	86 (51.49)	28 (62.23)	129 (50.20)		
Age No (%)	60 ~ 69 years	36 (21.55)	10 (22.22)	60 (23.34)	1.265^b^	0.531
	70 ~ 79 years	62 (37.12)	20 (44.44)	106 (41.24)		
	≥80 years	69 (41.33)	15 (33.34)	91 (35.42)		
Marital status No (%)	Married	109 (65.29)	31 (68.88)	166 (64.59)	5.201^(1)^	0.267
	Not Married	58 (34.71)	14 (31.12)	91 (35.41)		
Number of children No (%)	Two or more children	102 (61.09)	32 (71.12)	203 (79.00)	15.049^b^	0.001
	One children	5 (2.99)	3 (6.66)	7 (2.72)		
	No children	60 (35.92)	10 (22.22)	47 (18.28)		
Medical expense payment method No (%)	Urban medical insurance	45 (26.94)	11 (24.44)	104 (40.46)	15.207^a^	0.019
	Employee medical insurance	54 (32.33)	13 (28.88)	71 (27.62)		
	Out-of-Pocket	60 (35.92)	20 (44.44)	79 (30.73)		
	Other	8 (4.81)	1 (2.24)	3 (1.19)		
Occupation before disability No (%)	Farmer	61 (36.52)	13 (28.88)	128 (49.80)	12.473^a^	0.052
	Self-employed	67 (40.11)	19 (42.22)	73 (28.40)		
	Public institution staff	29 (17.39)	10 (22.22)	42 (16.34)		
	Other	10 (5.98)	3 (6.68)	14 (5.46)		
Education Level No (%)	Elementary School	83 (49.70)	18 (40.00)	85 (33.07)	12.992^b^	0.002
	Middle School	49 (29.34)	18 (40.00)	75 (29.18)		
	High School	17 (10.17)	5 (11.11)	78 (30.35)		
	College and Above	18 (10.79)	4 (8.89)	19 (7.40)		
Number of Diseases No (%)	1	51 (30.53)	18 (40.00)	97 (37.74)	0.123^b^	0.94
	2	52 (31.13)	15 (33.33)	79 (30.73)		
	3	21 (12.57)	2 (4.44)	30 (11.67)		
	>3	32 (25.77)	10 (22.23)	51 (19.86)		
Primary Disease Causing Disability No (%)	Respiratory System	46 (27.54)	16 (35.55)	66 (25.68)	12.343^a^	0.055
	Circulatory System	49 (29.34)	3 (6.66)	62 (24.12)		
	Nervous System	57 (34.13)	18 (40.00)	93 (36.18)		
	Other Systems	15 (8.99)	8 (17.79)	36 (14.02)		
Barthel Index Score m ± s		59.37 ± 18.79	62.89 ± 19.55	58.07 ± 18.54	1.317^c^	0.269
Degree of Disability No (%)	Mild disability	68 (40.71)	20 (44.44)	97 (37.74)	1.836^b^	0.399
	Moderate disability	62 (37.12)	20 (44.44)	104 (40.46)		
	Severe disability	37 (22.17)	5 (11.12)	56 (21.80)		
Duration of Disability No (%)	<1 year	120 (71.85)	34 (75.55)	139 (54.08)	10.820^b^	0.004
	1 ~ 2 years	19 (11.37)	3 (6.66)	99 (38.52)		
	≥3 years	28 (16.78)	8 (17.79)	77 (7.40)		
Monthly hospitalization frequency No (%)	1 time	40 (23.95)	8 (17.77)	72 (28.01)	5.965^b^	0.051
	2 times	38 (22.75)	10 (22.22)	50 (19.45)		
	≥3 times	89 (53.30)	27 (60.01)	135 (52.54)		
Understanding of disability No (%)	No	25 (14.97)	9 (20.00)	36 (14.00)	0.834^b^	0.659
	Partial	109 (65.26)	27 (60.00)	164 (63.81)		
	Full	33 (19.77)	9 (20.00)	57 (22.19)		
Source of income No (%)	Pension	107 (64.07)	27 (60.00)	138 (53.69)	6.422^a^	0.17
	Living with family	41 (24.55)	10 (22.22)	69 (26.84)		
	Other	19 (11.38)	8 (17.78)	50 (19.47)		
Monthly hospitalization days No (%)	<3 days	32 (19.16)	7 (15.55)	57 (22.17)	5.965^a^	0.051
	3-5 days	57 (33.72)	22 (48.88)	116 (45.13)		
	>5 days	78 (46.68)	16 (35.57)	84 (32.70)		
Living situation No (%)	Living with family	88 (52.69)	29 (64.44)	173 (67.31)	11.616^a^	0.002
	Other	21 (12.58)	11 (24.45)	25 (9.74)		
	Living alone	58 (34.73)	5 (11.11)	59 (22.95)		
Primary caregiver No (%)	Spouse	46 (27.54)	16 (35.55)	75 (29.18)	16.012^a^	0.014
	Children	52 (31.13)	11 (24.44)	115 (44.74)		
	Grandchildren	68 (40.71)	17 (37.77)	64 (24.90)		
	Other	1 (6.20)	1 (2.24)	3 (1.18)		
Frequency of visits from relatives or friends No (%)	0times/week	9 (5.38)	4 (8.88)	20 (7.78)	5.471^b^	0.065
	1time/week	30 (17.96)	14 (31.11)	36 (14.00)		
	2times/week	33 (19.76)	9 (20.00)	61 (23.73)		
	≥3times/week	95 (56.90)	18 (40.01)	140 (54.49)		
Self-rated family harmony No (%)	Not harmonious	15 (8.98)	2 (4.44)	10 (3.89)	18.682^b^	0.001
	Fairly harmonious	56 (33.53)	2 (4.44)	70 (27.23)		
	Harmonious	96 (57.49)	41 (91.12)	177 (68.88)		
Self-rated economic pressure No (%)	Not pressure	69 (41.31)	18 (40.00)	75 (29.18)	9.694^b^	0.008
	Some pressure	69 (41.31)	22 (48.88)	117 (45.52)		
	High pressure	29 (17.38)	5 (11.12)	65 (25.30)		
Frequency of communication with family No (%)	0 times/day	11 (6.58)	5 (11.11)	23 (8.94)	3.596^b^	0.385
	1 time/day	25 (14.97)	7 (15.55)	41 (15.95)		
	2 times/day	21 (12.57)	11 (24.44)	37 (14.39)		
	≥3 times/day	110 (65.88)	22 (48.90)	156 (60.72)		
Community support No (%)	Almost none	116 (69.46)	38 (84.44)	187 (72.76)	3.764^b^	0.152
	Rarely	27 (16.16)	4 (8.88)	33 (12.84)		
	Sometimes	17 (10.17)	1 (2.96)	32 (12.45)		
	Often	6 (3.59)	1 (2.96)	2 (0.78)		
	Always	1 (0.62)	1 (2.96)	3 (1.17)		

a Chi-square test.

b Rank sum test.

c Analysis of variance.

### 3.4 Multivariate analysis results of fatalism profiles in community-dwelling disabled elderly individuals

Given that the latent profile analysis did not reveal significant ordinal relationships among dimensions, unordered multinomial logistic regression was employed to identify the influencing factors for different fatalism profiles among community-dwelling disabled elderly individuals.

Before conducting the multivariate analysis, collinearity diagnostics were performed on the variables identified as statistically significant in the univariate analysis. The results of these diagnostics indicated that the tolerance for each variable was greater than 0.1, and the variance inflation factors (VIF) were below 10. These findings suggest that multicollinearity among the variables was not a concern in the analysis.

This study examined the factors influencing fatalism profiles among disabled elderly individuals. The results showed that compared to disabled elderly individuals living alone, those living with family (OR = 0.067, 95% CI [0.016–0.277]) or others (OR = 0.246, 95% CI [0.069–0.877]) were more likely to be classified as Class 3. Having two or more children was linked to a lower chance of being classified as Class 1 compared to having no child (OR = 0.380, 95% CI [0.223–0.648]). Individuals with a middle school education were more likely to be classified as Class 1 compared to those with higher education (OR = 2.670, 95% CI [1.015–7.024]). Those with a disability duration of 1 to 2 years had a higher likelihood of being classified as Class 1 compared to those with longer disability durations (OR = 2.958, 95% CI [1.313–6.666]). Finally, elderly individuals with no economic pressure were more likely to be classified as Class 3 compared to those with high economic pressure (OR = 0.415, 95% CI [0.213–0.810]).

**Figure 3:**
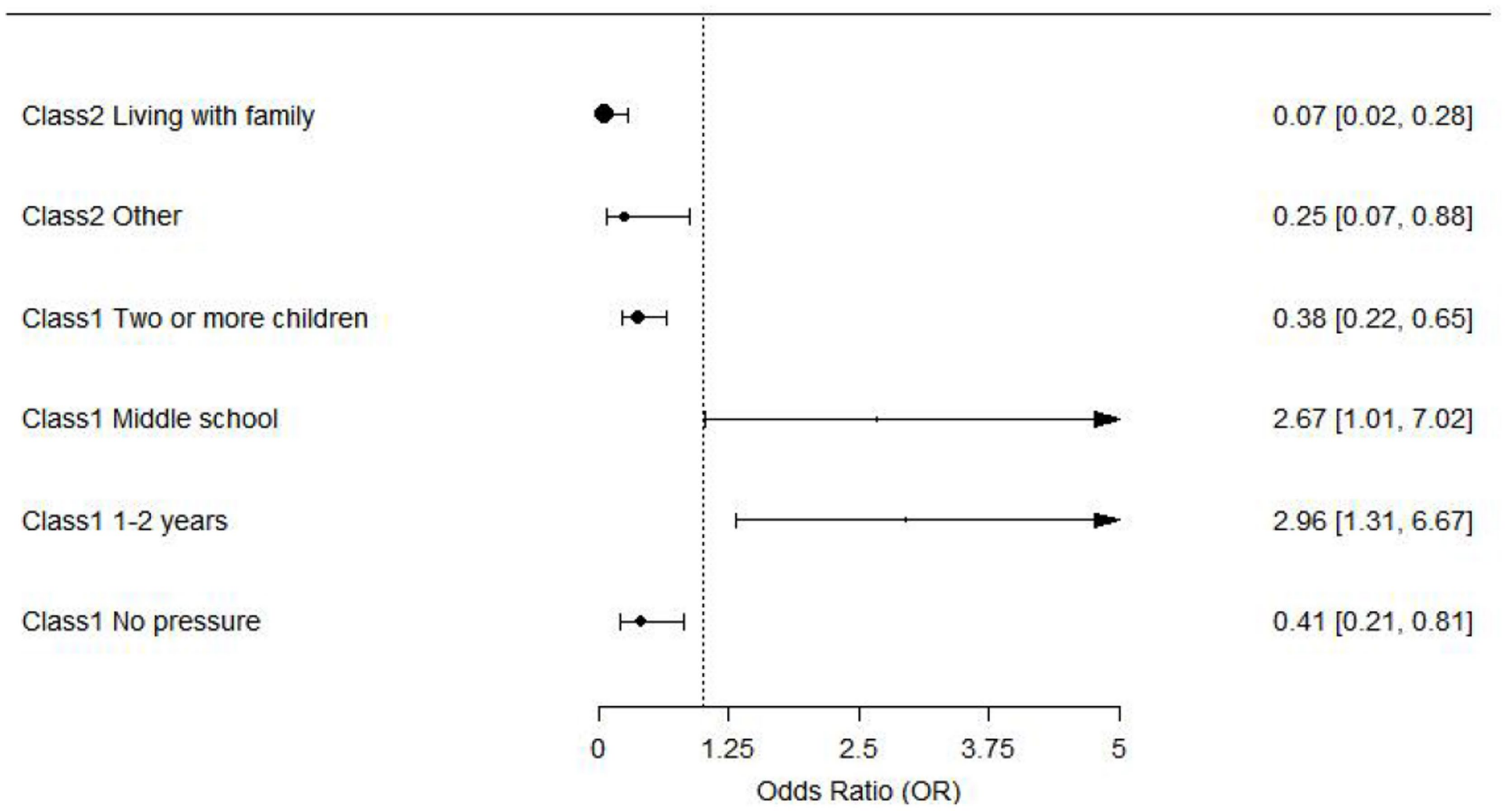
Forest plot of factors influencing fatalism profiles among community-dwelling disabled elderly individuals.

In the study, only results with statistical significance are presented, while detailed regression analysis of the factors influencing fatalism profiles among community-dwelling disabled elderly individuals is provided in Table 3. This table includes the odds ratios (OR) along with their 95% confidence intervals (CIs) for each factor analyzed in relation to fatalism. The results are further visualized in the accompanying forest plot (Figure 3), offering an intuitive graphical representation of these findings.

**Table 3:** Regression analysis of the three latent categories of fatalism in community-dwelling elderly individuals with disabilities.

Category	Variable	Item	B	Standard error of B	Wald chi-square value	*P*	*OR*	95%CI
Class 2	Living situation	Living with family	−2.699	0.722	13,973	<0.001	0.067	0.016–0.277
		Other	−1.404	0.649	4.671	0.031	0.246	0.069–0.877
Class 1	Number of children	Two or more children	−0.968	0.273	12.627	<0.001	0.380	0.223–0.648
	Education level	Middle school	0.982	0.493	3.961	0.047	2.670	1.015–7.024
	Duration of disability	1–2 years	1.085	0.415	6.846	0.009	2.958	1.313–6.666
	Self-rated economic pressure	No pressure	−0.880	0.341	6.650	0.010	0.415	0.213–0.810

Class 3 as the reference group.

## 4 Discussion

### 4.1 Analysis of the potential profiles of fatalism in community-dwelling disabled elderly

Fatalism significantly influences the later stages of life for disabled elderly individuals, yet it remains underexplored in current research. Defined as a belief in the uncontrollability of fate, fatalism can adversely affect psychological well-being and quality of life, leading to diminished agency and increased emotional distress (Valenti and Faraci, 2022). Despite its potential impact, existing literature provides limited insights into this issue, highlighting the need for further investigation.

This study used latent profile analysis to identify three distinct symptom categories based on the fatalistic attitudes of community-dwelling disabled elderly individuals. These categories provide insights into how different groups cope with life’s challenges and pressures, enriching the understanding of fatalism in elderly populations.

By identifying groups with distinct fatalistic traits, we can better address their psychological needs and design tailored interventions. For those with strong fatalistic attitudes, support should focus on improving perceptions of fate and reducing reliance on fatalism. Balanced groups may benefit from interventions that enhance their sense of control and foster positivity, while those with weaker fatalistic views can gain from strengthened social support networks. These classifications highlight the importance of addressing fatalism in elderly care to improve mental health and quality of life.

### 4.2 Analysis of factors shaping fatalism profiles in community-dwelling disabled elderly

#### 4.2.1 The impact of living situation on fatalism

The results showed that disabled elderly individuals living alone were more likely to be classified as Class 2, while those cohabiting with family or others, such as colleagues or friends, tended to fall into Class 3. This may be due to the greater daily and emotional support available to those living with others (Long et al., 2023). Cohabitants provide assistance with meals, hygiene, and daily activities, reducing stress and uncertainty while offering psychological support through regular interactions (Zheng et al., 2023). This stable support system enhances life satisfaction and resilience, fostering a more positive outlook and moderate fatalism, aligning their psychological state with Class 3.

In contrast, elderly individuals with disabilities living alone are more likely to be classified into Class 2, which may be related to their unique lifestyle and behavior patterns. Living alone and lacking assistance from others, these elderly individuals must handle many daily tasks on their own, such as shopping, cooking, and personal care. This self-care requirement encourages the development of some degree of independence but also brings greater life stress and physical and mental burdens (Gao et al., 2021). The lack of support from family members or others makes their anxiety more prominent in daily life, leading to higher anxiety and pessimism when facing difficulties. Additionally, elderly individuals living alone may be more inclined to reduce social interactions or outdoor activities, which increases their sense of loneliness and creates a more closed-off lifestyle (Scarantino et al., 2022; Zhang and Dong, 2022). Over time, the lack of social support and emotional interaction may further exacerbate their negative views on life, influencing their classification.

Based on the analysis of influencing factors, practical nursing interventions can be implemented for elderly individuals with disabilities in different living environments to improve their psychological health and quality of life. For elderly individuals living with family members or others, nursing interventions should focus on enhancing internal family interactions and support. Family members play a crucial role in the daily care of disabled elderly individuals (Li et al., 2023). Therefore, encouraging family members to participate more in daily tasks and psychological support for the elderly is vital. Nursing staff can facilitate regular family support group meetings or training to help family members understand the elderly individual’s care needs, promote emotional communication, and reduce family conflict and stress caused by the elderly person’s disability. At the same time, encouraging family members to provide more emotional companionship can help the elderly maintain a positive mindset and alleviate anxiety and depression (Yang et al., 2023).

#### 4.2.2 Impact of the number of children on fatalism profiles

In this study, the number of children significantly affects the symptom characteristics of fatalism in disabled elderly individuals. Specifically, elderly individuals with two or more children are more likely to be classified into Class 3. The reasons for this can be analyzed as follows: for disabled elderly individuals with two or more children, mutual support and care among family members play a crucial role in their psychological well-being. These elderly individuals usually receive more comprehensive daily care, such as assistance with daily tasks, medical care, and emotional companionship from their children. The interactions and cooperation between children provide more emotional support, significantly reducing feelings of anxiety and loneliness in the elderly (Shulyaev et al., 2024). This emotional connection helps them maintain a more positive attitude when facing the challenges of disability. This stable support system helps reduce fatalism and pessimism, making them more likely to be classified into Class 3, characterized by lower fatalism and higher optimism.

In contrast, disabled elderly individuals with no children or fewer children face a more difficult living situation (Du et al., 2024). First, the lack of direct care and companionship from children makes the emotional support sources for these elderly individuals relatively limited, leading to increased feelings of loneliness and pressure (Gan et al., 2023). Especially as the daily needs of disabled elderly individuals increase, the lack of children or having fewer children makes it difficult for them to receive timely and sufficient help and care. Moreover, without close family members to rely on, elderly individuals living alone or with non-immediate relatives often become more dependent on external services or social support. However, the lack of or instability in the social support system may not effectively replace the emotional support and daily care provided by the family (Kobayashi et al., 2023). Therefore, these elderly individuals are more likely to experience stronger fatalism and pessimism when facing life pressures and are more likely to be classified into Class 1, characterized by higher fatalism and lower optimism.

To effectively improve the psychological health and quality of life of disabled elderly individuals in families with varying numbers of children, targeted intervention measures should include the following aspects: For elderly individuals with multiple children, it is essential to encourage cooperation and coordination among the children to ensure balanced distribution of daily care and emotional support. For disabled elderly individuals with no children or fewer children, greater focus should be placed on strengthening community support systems, providing regular home care, health check-ups, meal delivery services, and other services to relieve the elderly’s daily pressures and ensure proper care (Fukumoto et al., 2023). At the same time, elderly individuals should be encouraged to engage in social interactions with other community members, participate in community activities and volunteer services, increase social opportunities, and establish external support networks.

#### 4.2.3 Impact of educational level on fatalism profiles

In this study, elderly individuals with a middle school education were more likely to be classified into Class 1, reflecting the specific impact of education level on psychological health and life attitudes. Compared to elderly individuals with other levels of education, those with a middle school education tend to exhibit stronger fatalistic beliefs and more pessimistic emotions. This phenomenon may stem from the cognitive and social adaptation differences associated with the education level represented by a middle school education. Compared to individuals with higher education, those with a middle school education typically have fewer cognitive resources and problem-solving skills (Stephens et al., 2023). Therefore, when faced with life challenges such as disability, they may find it difficult to adopt effective coping strategies, leading to emotional distress and negative cognitions.

From a social support perspective, elderly individuals with a middle school education are often situated in more fragile social networks. While they may sometimes rely on relatives or neighbors, this support is often loose and unstable (Li et al., 2022). In contrast, individuals with elementary or lower education may depend more on family or long-term community care, which provides them with a greater degree of stability. Those with a high school education or above are more likely to rely on self-regulation and independent problem-solving abilities, as they possess stronger social interaction skills and resource access capabilities, enabling them to better cope with life challenges (Chen et al., 2020). Therefore, elderly individuals with a middle school education often lack both the support and capabilities needed to cope effectively, making them more prone to stronger fatalistic beliefs and pessimism (Mesas et al., 2020).

To address the needs of elderly individuals with a middle school education, community interventions should focus on enhancing their social support and life adaptation abilities. Services such as regular health check-ups, daily care, and psychological counseling can provide more support for this group. In particular, community efforts should strengthen attention to their emotional companionship and psychological guidance, offering personalized assistance to alleviate feelings of loneliness and pessimism. Furthermore, community activities and social interactions should be promoted to encourage elderly individuals with a middle school education to participate, enhancing their social engagement and reducing feelings of isolation.

#### 4.2.4 Impact of duration of disability on fatalism profiles

In this study, community-dwelling disabled elderly individuals with a disability duration of 1–2 years were more likely to be classified as Class 1, characterized by stronger fatalistic beliefs and pessimistic emotions. This finding underscores the unique psychological impact of disability duration. Compared to individuals with shorter disability durations (less than 1 year) or longer durations (more than 2 years), those in the 1–2 year range appear to be in a critical transitional phase of psychological adjustment, facing heightened emotional fluctuations and psychological distress.

From an adaptation perspective, individuals with a shorter duration of disability may still be in the “shock and denial” phase, where the full acknowledgment of their condition is incomplete, and psychological defense mechanisms mitigate some negative emotions (Pahor et al., 2020). Conversely, individuals with longer disability durations may have undergone prolonged psychological adjustment and lifestyle restructuring, allowing them to transition to the “acceptance and adaptation” phase, where they gradually accommodate the changes in their daily lives and find new life priorities. However, those with a disability duration of 1–2 years are often in a “midway dilemma” phase. At this stage, the decline in physical function has already caused significant disruptions to daily life, but psychological acceptance remains incomplete (Ogawa et al., 2021). This discordance between their psychological state and lived experience may lead to feelings of helplessness and pessimism.

From a social support perspective, individuals in this transitional phase may also encounter instability in their support systems. During the initial stages of disability, family members and community services typically provide substantial attention and assistance (Jadhav et al., 2021). However, over time, this support often diminishes, particularly during the 1–2 year “transition period, “when familial and community support tends to normalize, and the individual’s adaptive capacity is not yet fully developed, potentially increasing feelings of isolation. Additionally, individuals in this phase may face challenges related to shifts in their social roles, such as losing work capacity or reducing social engagement, further exacerbating psychological stress (Putek et al., 2023).

To address the needs of community-dwelling disabled elderly individuals with a disability duration of 1–2 years, a comprehensive intervention approach integrating family support and professional medical care is essential. Families should encourage regular communication to monitor the elderly’s psychological state, facilitate emotional expression, and alleviate negative feelings (Basoglu et al., 2022). Creating a positive atmosphere through effective communication and shared caregiving responsibilities, as well as engaging the elderly in household activities or interactions with grandchildren, can enhance their sense of participation and purpose.

From a medical perspective, healthcare providers should offer specialized psychological support, including mental health assessments, counseling referrals, and individualized rehabilitation plans. These plans should include physical activity training, occupational therapy, and chronic disease management education to improve self-efficacy and reduce the psychological impact of health issues. Collaboration among general practitioners, nurses, rehabilitation therapists, and social workers is critical for ongoing support and regular follow-ups to monitor both physical and psychological well-being. By combining family support with professional care, psychological distress can be alleviated, quality of life improved, and the ability to cope with disability strengthened during this transitional phase (Kalnina et al., 2024).

#### 4.2.5 Impact of self-rated economic pressure on fatalism profiles

The analysis revealed that disabled elderly individuals with significant economic pressure were more likely to be classified in Class 1 than those without economic pressure. This finding indicates that elderly individuals with better economic conditions are less likely to exhibit fatalism and are more likely to maintain an optimistic outlook on life. Conversely, those experiencing economic hardship are prone to negative emotions and stronger fatalistic beliefs. Economic challenges can exacerbate feelings of helplessness and pessimism, leading to a more fatalistic view of life (Sigurdardottir et al., 2019). This underscores the importance of economic support in enhancing the psychological well-being of disabled elderly individuals.

From a mechanistic perspective, elderly individuals under economic pressure often experience physical and mental exhaustion from the ongoing strain of daily expenses and medical costs (Li et al., 2024). When household income is insufficient to cover substantial medical and caregiving expenses, these individuals are more likely to feel pessimistic about the future, potentially abandoning active engagement with life (Rodriguez-Pereira et al., 2020). Economic constraints also limit participation in social activities, such as community events or educational programs, further reducing social support and emotional interactions, which exacerbates loneliness and psychological stress.

To address the needs of elderly individuals under economic pressure, a multi-faceted approach to intervention is essential. From a social support perspective, governments and communities should strengthen economic aid policies, such as increasing disability subsidies or expanding social security coverage for low-income groups, ensuring that basic living needs are met. Communities can also establish special assistance funds to provide temporary relief for individuals with urgent economic needs. Additionally, partnerships with non-governmental organizations and philanthropic initiatives can deliver medical subsidies, material support, and opportunities for free participation in community activities.

From a healthcare perspective, medical professionals should deliver targeted psychological support for elderly individuals facing economic pressure. This includes providing regular counseling to alleviate anxiety and assisting them in accessing medical aid programs through local governments or charitable organizations to ease the financial burden of health issues. By addressing economic stressors through comprehensive social, family, and healthcare interventions, it is possible to improve the mental health and overall well-being of elderly individuals experiencing economic hardship.

## 5 Conclusion

This study, through latent profile analysis, identified three distinct fatalism tendencies among community-dwelling disabled elderly individuals: high fatalism and pessimistic tendency, moderate fatalism and low optimism Tendency, low fatalism and relatively optimistic tendency. These classifications offer new perspectives for understanding and addressing the psychological states of disabled elderly individuals and lay a foundation for developing personalized intervention strategies.

The findings emphasize the heterogeneity of fatalism tendencies among disabled elderly individuals and highlight the associations between fatalism tendencies and their living conditions. These results provide important insights for designing targeted psychosocial interventions, particularly in enhancing the psychological well-being and quality of life of disabled elderly individuals.

Based on the results of this study, future intervention strategies should consider the specific needs of disabled elderly individuals with varying fatalism tendencies to achieve more precise care and management. Furthermore, the findings offer new directions for future research, particularly in exploring the influencing factors of fatalism tendencies and the effectiveness of interventions.

In conclusion, this study not only advances the understanding of fatalism tendencies among community-dwelling disabled elderly individuals but also provides an empirical foundation for the development of effective health promotion and psychological support strategies.

## 6 Limitations

This study was limited to examining the fatalism profiles of disabled elderly individuals within different regions of Sichuan Province, which may introduce a risk of regional bias. Future research should aim to expand the geographical scope and sample size to achieve a more comprehensive understanding of fatalism among disabled elderly populations. Additionally, this study was limited by its cross-sectional design, which restricts the ability to establish causal relationships between the variables. The findings are correlational and should not be interpreted as causal. Future research with longitudinal designs is recommended to validate the temporal relationships between the variables and better understand their causal pathways.

## Data Availability

The raw data supporting the conclusions of this article will be made available by the authors, without undue reservation.
